# How the COVID-19 Pandemic affected the Bereavement Process

**DOI:** 10.1192/j.eurpsy.2023.489

**Published:** 2023-07-19

**Authors:** B. Calado Araújo, D. Krupka

**Affiliations:** Centro Hospitalar Universitario do Algarve, Faro, Portugal

## Abstract

**Introduction:**

The COVID-19 pandemic has caused millions of deaths worldwide. However, unlike a natural disaster, it has also affected end-of-life care and funeral rites through social restrictions.

**Objectives:**

Understand how the COVID-19 pandemic affected the grieving process.

**Methods:**

A PubMed literature search for all relevant studies was conducted used terms such as “prolonged gried disorder”, “grief”, “mourning”, “bereavement” and “COVID-19”. The articles were selected after two different analysis, a first one based on their titles and abstracts and a second one based on their full texts.

**Results:**

Based on the evidence extracted from these articles, it is clear that the COVID-19 pandemic has had an important effect on the bereaved population. More specifically, several articles found there to be an increase in the prevalence of severe grief symptoms caused by deaths that occur during the pandemic period, regardless of cause of death (Eisma and Tamminga 2020, Tang and Xiang 2021, Breen, Mancini et al. 2022, Downar, Parsons et al. 2022, Gang, Falzarano et al. 2022). These results were explained by the disruption the grieving cycle due to social restriction which occurred during the pandemic period. These restrictions prevented the bereaved person from saying goodbye and being present at the time of death, holding mourning ceremonies, as well as having the needed social support during the mourning period (Goveas and Shear 2020, Kokou-Kpolou, Fernández-Alcántara et al. 2020, Mortazavi, Assari et al. 2020, Tang and Xiang 2021, Downar, Parsons et al. 2022). Regarding causes of death, the result were inconsistent, namely one study found to be higher grief levels associated with COVID-19 deaths when compared to natural causes, but not when compared to unnatural deaths, such as accidents and homicides (Gang, Falzarano et al. 2022). While another study found COVID-19 deaths caused are severe grief reactions when compared to natural deaths (Eisma and Tamminga 2022).

**Conclusions:**

The COVID-19 pandemic has increased the prevalence of severe grief symptoms and therefore it is important for the scientific community to be sensitized to this effect. However, there is still a lack of studies concerning this theme, which are essential to define a course of action.

**Disclosure of Interest:**

None Declared

